# Comparative Radiographic Assessment of Root Canal Obturation Quality: Manual Verses Rotary Canal Preparation Technique

**Published:** 2014-06

**Authors:** Ghafoor Robia

**Affiliations:** The Aga Khan University Hospital Karachi, Pakistan

**Keywords:** Root canal preparation, obturation quality, rotary and manual canal preparation techniques

## Abstract

**Objective:**

To compare the technical quality of root canal obturation in relation to rotary and manual root canal preparation techniques.

**Study Design:**

Cross sectional, analytical study.

**Place and Duration of Study:**

The Aga Khan University Hospital Karachi from January till December 2011.

**Methodology:**

Data was retrospectively retrieved by periapical radiographs of 60 root canal treated molars for assessment of obturation quality. Data was divided into two groups, 30 radiographs in each group. Group 1 and 2 teeth were prepared with rotary and manual canal preparation technique respectively followed by obturartion with cold lateral condensation technique. Postobturation radiographs were assessed to evaluate technical quality of obturation in term of length, density and taper of root filling. Mann Whitney-U and Chi- square test was applied to compare the difference in technical quality of obturation between two canal preparation techniques.

**Results:**

Amongst Thirty molar prepared with rotary instruments, 27 had achieved acceptable length, 25 had adequate density and 27 had achieved adequate taper. Amongst 30 molars prepared manually, 18 had achieved acceptable length, 14 had adequate density and 12 had adequate taper.

**Conclusion:**

Radiographic technical quality of the obturation (length, density and taper) was better with rotary technique as compared to manual canal preparation technique.

## INTRODUCTION

The prognosis of root canal treatment (RCT) depends on many variables; amongst them is the technical quality of the root filling (1, 2). During the last decade, endodontic therapy went through a fascinating development. The introduction of operating microscope, rotary nickel titanium instruments, and Protaper enabled the practitioner to better shape the root canal system (3).

The importance of maintaining the original shape of a root canal during and after cleaning and shaping in order to promote periapical healing in endodontic cases has been demonstrated in several studies (4-6). The clinician’s inability to maintain the original shape and to develop the proper taper of canals can result in procedural errors such as ledges and perforations. It has been shown that endodontic treatment success is dependent both on the quality of the obturation and the final restoration (7).

The quality of the endodontic obturation is usually evaluated using radiographic images upon completion. Additionally, during the root canal preparation and obturation phases of treatment, clinical criteria can be identified that are essential for achieving an adequate root canal obturation (8-10).

Several variables affect the technical quality of root fillings, such as the length of the filling material in relation to the radiographic apex, the density of the root filling material (presence of voids), the taper of the canal. Methods used to evaluate the technical outcome of RCT have been based mostly on radiographic evaluation (11).

Root fillings placed within 0–2 mm of the radio- graphic apex are associated with less post-treatment disease than those that are filled with a distance more than 2 mm from the radiographic apex (12, 13). Studies have reported that the length of the root filling, relative to the radiographic apex, significantly affected the outcome of RCT with 87-94% healing rates associated with root filling ending 0–2 mm from the radiographic apex. Lower healing rates were associated with ‘short’ root fillings ending more than 2 mm from the radiographic apex (68–77.6%) and with long fillings extruding beyond the apex (75–76%) (14, 15).

The correlation between density of the root filling and prognosis is not as clear as the proximity of the root filling to the radiographic apex. Several studies have reported no difference in prognosis between adequately and in adequately compacted root fillings (14, 16). Whilst others have found that a root filling that is less dense and non-homogenous will have a negative impact on the outcome (17, 18).

Canal preparation must flow and progressively narrow in an apical direction starting at the orifice and moving apically, every cross-sectional diameter of the filling material should decrease with the smallest cross-sectional diameter at the apical terminus of the canal (19). A continuous taper in the apical third of the shaped canal creates a resistance form for gutta- percha and reduces the potential for overextensions (20).

Procedural errors compromise canal cleaning and shaping and result in incomplete root filling, which jeopardizes the outcome of the treatment (21). Clinicians generally believe that endodontic procedural errors, such as underfilling, overfilling, separated instruments, root perforations and ledge formation, are the direct cause of endodontic treatment failure. However, procedural errors by themselves do not jeopardize the outcome of treatment unless a concomitant infection is present (22).

Several endodontic epidemiological studies had been carried out in different population groups to assess technical quality of root filling in relation to performer experience performed by undergraduate students using standard step back canal preparation technique followed by lateral condensation for canal obtuartion (23-26).

Since yet, limited studies has been conducted to relate quality of obturation with different canal instrumentation techniques. Therefore, the aim of the present study was to evaluate the obturation quality between manual and rotary instrumentation technique in clinical practice.

## METHODOLOGY

It was a descriptive cross sectional study design, held at The Aga Khan University Hospital Dental clinic from January 2011 till September 2011. The data used in the current study consisted of a sample of periapical radiographs of patients who received root canal treatment at the Aga Khan University Hospital as a routine investigation with in practice and were not specifically taken for the study. An effort was made to exclude most of the radiographs with superimposed canal fillings or over-projection of anatomical structures, to eliminate the possibility of radiographic misinterpretation. All permanent maxillary and mandibular molars in which root canal treatment were indicated either clinically or recommended due to elective endodontics were included in the study. All molars with severely curved roots, having sclerosed canals, tooth require endodontic retreatment or tooth with apical root resoption were excluded from study. A total sample of 60 periapical radiographs of maxillary and mandibular molars were identified on the basis of non probability, purposive sampling technique. They were equally divided into two groups, using alternate group allocation. Group-I (n=30) molars were prepared manually by Consultants and Residents of Operative Dentistry. Group-II (n=30) molars were prepared by protaper Ni Ti system up to size 30 using a crown down preparation technique by Consultants and Residents of Operative Dentistry. All molars of Group-I were obturated by cold lateral condensation gutta percha technique using calcium hydroxide based sealer, while all molars of Group-II were obturated with gutta percha points of protaper system (greater taper) using lateral calcium hydroxide based sealer. Postoperative Periapical radiographs of root canal treated molars were obtained immediately after the obturation using paralleling device.

Evaluating the technical quality of root fillings was based on the preoperative, working length determination and post- operative radiographs (mesial and distal angulated radiographs were included for multi-rooted teeth). The radiographs were evaluated independently by two senior endodontists (with minimum experience of 8 years). Films were examined using handheld X-ray film viewer with magnifying lens that could be moved in different angulations for varying magnification (Meta Biomed Co., LTD, Cheongju City, Korea). The results were compared, and a final evaluation was agreed. In case of disagreement, the two examiners discussed the case to reach a consensus.

The quality of the root fillings was evaluated according to the distance between the end of the filling and the radiographic apex, the density of the filling and the taper of the root filling using the criteria of Barrieshi- Nusair *et al*. (25) (Table 1) and on the basis of this criteria a scoring system (T-Score) was made to score technical quality of root filling (Table 2).

**Table 1:** Criteria used to assess radiographic of root filling

Parameter	Criteria	Define quality of root filling

Length of root filling	Adequate	Root filling ending ≤ 2 mm from radiographic apex
	Overfilling	Root filling beyond the radiographic apex
	Short filling	Root filling >2 mm from radiographic apex
	Flush	Root filling at the radiographic apex
Density of root filling	Adequate	No voids present in the root filling or between root filling and root canal walls
	Inadequate	Voids present in the root filling or between root filling and root canal walls
Taper of root filling	Adequate	Consistent taper from the orifice to the apex
	Inadequate	No consistent taper from the orifice to the apex

**Table 2:** T-score

Score 3 - Obturated canals have all three qualities of ideal Obturation.
Adequately filled (with in 2mm from radiographic apex).
Smooth coronal apical taper.
No voids.
Score 2 - Obturated canals have any two qualities of ideal obturation.
Score 1 - Obturated canals have any one quality of ideal obturation.
Score 0 - Obturated canals have no quality of ideal obturation.

For statistical analysis, the tooth was considered as a unit with the highest score of all roots contributing the score. ‘Acceptable’ filling quality was defined as adequate length, density and taper with the absence of any procedural error. Inter-examiner agreement was measured by Cohen’s kappa (k) values. The analysis of the data was performed using SPSS 14.0 for Windows (SPSS Inc., Chicago, IL, USA). Sample means and their standard errors were used to describe every item listed on the evaluation form. The chi-squared statistic was used to compare obturation quality of root filling in term of length, density and taper in relation to canal preparation technique.

The overall assessment of obturation quality (length, density and taper) between two canal preparation technique were analyzed by applying Mann Whitney-U Test. A *P*-value <0.05 was considered statistically significant.

## RESULTS

The k-value for inter-examiner reliability was 0.94 for length of root filling, 0.96 for density, 0.97 for taper. Amongst thirty molars prepared with rotary instruments (Protaper system), 27 (90%) had obturation up to acceptable length, 25 (83.3%) had adequate density and 27 (90%) had adequate taper. Whereas amongst thirty molars prepared manually 18 (60%) had obturation of acceptable length, 14 (46.7%) had adequate density 12(40%) had adequate taper (Table 3).

**Table 3:** The length, density and taper in relation to canal preparation technique

Obturation Quality		Manual canal preparation n=30 (%)	Rotary canal preparation n=30 (%)	Totaln=60(%)

**Length of root filling**	Adequately filled	18 (60)	27 (90)	45 (75)
	Under filled	10 (33.3)	1 (3.3)	11 (18.3)
	Over filled	2 (6.7)	2 (6.7)	4 (6.7)
**Density of root filling**	Inadequate	16 (53.3)	5 (16.7)	21 (35)
	Adequate	14 (46.7)	25 (83.3)	39 (65)
**Taper of root filling**	Adequate	12 (40)	27 (90)	39 (65)
	Inadequate	18 (60)	3 (10)	21 (35)

Data is expressed as n= number of patients (%).

Assessment of overall technical quality of root filling (using T-Score) revealed statistically significant difference (*p*<0.001) in relation to canal preparation technique (Table 4) but no statistically significant difference (*p*=0.371) was observed in relation to intra arch tooth positioning (Table 5) and even gender dimorphism had no statistically significant value (*p*=0.163) on technical quality of root filling (Table 6). There was statistically significant difference in length of root filling (p < 0.001), density of root filling (*p*<0.001) and taper of root filling (*p*<0.001) in relation to canal preparation technique (Table 7).

**Table 4:** Assessment of obturation quality of root canal filling in relation to canal preparation technique

Preparation technique		No of tooth	Mean rank	*p* value

T-Score	Manual preparation	30	21.85	0.001
	Rotary preparation	30	39.15	

Mann Whitney U test, *p* value<0.001.

**Table 5:** Assessment of obturation quality of root canal filling in relation to intra arch tooth position

Intra arch tooth position		No of tooth	Mean rank	*p* value

T-Score	Mandibular molars	33	31.63	0.371
	Maxillary molars	27	27.41	

Mann Whitney U test, *p* value=0.371.

**Table 6:** Assessment of obturation quality of root canal filling in relation to gender dimorphism

Gender dimorphism		No of tooth	Mean rank	*p* value

T-Score	Male	30	32.88	0.163
	Female	30	26.94	

Mann Whitney U test, *p* value=0.163.

**Table 7:** Comparison of technical quality of root filling in relation to canal preparation technique

Technical quality of root filling	Criteria for technical quality of root filling	Manual preparation	Rotary preparation	Chi-square value	df	*p* value

**Length of root filling**	Adequate	18	27	16.20	2	<0.001
	Under filled	10	1			
	Overfilled	2	2			
**Density of root filling**	Adequate	14	25	8.864	1	<0.001
	Inadequate	16	5			
**Taper of root filling**	Adequate	12	27	16.48	1	<0.001
	Inadequate	18	3			

## DISCUSSION

The k-values of 0.94, 0.96 and 0.97 in the ratings of root filling length, density and taper, respectively, indicate excellent agreement between the examiners because of well-defined criteria used. In current study obturation quality was compared with repcet to manual (Stainless Steel) and rotary (Protaper Ni Ti) canal instrumentation technique. Molars prepared manually with stainless steel files developed more procedural errors and the overall quality of obturation was scored less as compared to rotary canal preparation technique. Similarly Kleier DJ *et al.* conducted a study in order to compare clinical outcomes using a nickel titanium rotary and stainless steel hand file instrumentation technique. They found that when the rotary file technique was substituted for the hand file technique, appointment time for case completion was significantly decreased (*P*<0.001) and overall quality of Mandibular mesial root obturations were significantly increased (27).

In the present study, the percentage of root fillings with adequate length was 75% with manual canal preparation technique and 90% with rotary canal preparation technique. This frequency was superior to those reported by Er *et al*. (70%), Lupi-Pegurier *et al.* (39%), Chueh *et al.* (62%) and Eleftheriadis & Lambrianidis (63%) (23, 24, 28, 29).

Studies that addressed the lateral adaptation of the root filling as a criterion generally agreed that if void was present between the filling and the canal walls, the filling should be categorized as inadequate. Kirkevang *et al.*reported that inadequate density may lead to failure of RCT because of microleakage along the root filling (30).

Similarly, Eriksen & Bjertness stated that the prevalence of apical periodontitis was higher in root filled teeth with poor densities. The result of the present study indicated that adequate density without voids was achieved in 25 teeth (83.3%) and 14 teeth (46.7%) in case of rotary and manual canal preparation technique respectively similarly, Yoldas *et al.* reported adequate density without voids was 64% and Sagsen *et al.* reported 53% (31-33).

However, it is difficult to compare the studies as a result of differences in the sample size and change in methodology. Moreover, many studies (4, 5, 34) reported that when dental students used either hand or rotary nickel– titanium instruments, canals were prepared with less procedural errors and more successful treatment occurred compared to using conventional stainless steel instruments (34).

Studies evaluating the radiographic quality of RCT were mostly based on the evaluation of both the length and the density of the root filling (Helminen *et al.* 2000, Kirkevang *et al.* 2001, Lupi-Pegurier *et al.* 2002 and Dugas *et al*. 2003) (28, 30, 35, 36). However, the prepared root canal should be uniformly tapered from crown to apex.

The frequency of teeth with adequate taper of root filling in the present study was less (40%) with manual preparation but significantly higher in case of rotary preparation (90%). Overall adequate taper in the present study was (65%) almost comparable to the results of previous studies (23, 25, 33). This could be attributed to the highly subjective assessment of this variable radiographically.

All procedural errors cannot be depicted on radio- graphs. Over-instrumentation, for example, which may push pulp remnants and microorganisms beyond the apex causing acute apical periodontitis, can be detected by the use of radiographs only when it is followed by extrusion of filling material but not during previous stages of RCT (37).

Pettiette *et al.* and Gluskin *et al*. reported that when dental students used either hand or rotary nickel– titanium instruments, canals were prepared with less procedural errors and more successful treatment occurred compared to using conventional stainless steel instruments (5, 34).

Whilst the technical quality of root fillings, as portrayed by radiographs, is important for the outcome of the treatment, it may not reflect the quality of the treatment in general. The antiseptic and aseptic efforts during treatment, quality of canal preparation, materials used are amongst many prognostic factors that effect on treatment outcome.

## CONCLUSION

Overall quality of obturation was better with rotary canal preparation technique as compared to manual canal preparation technique (*p* value<0.001).
